# Estimating human leishmaniasis burden in Spain using the capture-recapture method, 2016–2017

**DOI:** 10.1371/journal.pone.0259225

**Published:** 2021-10-29

**Authors:** Ana María Humanes-Navarro, Zaida Herrador, Lidia Redondo, Israel Cruz, Beatriz Fernández-Martínez

**Affiliations:** 1 Hospital Clínico San Carlos, Madrid, Spain; 2 National Centre for Epidemiology, Instituto de Salud Carlos III (ISCIII), Madrid, Spain; 3 Network Biomedical Research on Tropical Diseases (RICET in Spanish), Madrid, Spain; 4 National Centre for Tropical Medicine, Instituto de Salud Carlos III (ISCIII), Madrid, Spain; 5 National School of Public Health, Instituto de Salud Carlos III (ISCIII), Madrid, Spain; 6 Consortium for Biomedical Research in Epidemiology and Public Health (CIBERESP), Madrid, Spain; University of Ostrava, CZECH REPUBLIC

## Abstract

Leishmaniasis is endemic and a mandatory reporting disease in Spain since 1982. However, between 1996 and 2014, surveillance on public health was decentralized and only some autonomous regions monitored the disease. The aim of this study is to estimate the incidence of leishmaniasis and to evaluate the extent of underreporting in Spain. A capture-recapture (CRC) study was conducted to calculate the incidence of human leishmaniasis using reports from the National Surveillance Network (RENAVE) and the Hospital Discharge Records of the National Health System (CMBD) for 2016 and 2017. During the study period, 802 cases were reported to RENAVE and there were 1,149 incident hospitalizations related to leishmaniasis. The estimated incidence rates through the CRC study were 0.79 per 100,000 inhabitants for visceral leishmaniasis (VL), 0.88 (cutaneous leishmaniasis (CL)) and 0.12 (mucocutaneous leishmaniasis (MCL)) in 2016 and 0.86 (VL), 1.04 (CL) and 0.12 (MCL) in 2017. An underreporting of 14.7–20.2% for VL and 50.4–55.1% for CL was found. The CRC method has helped us to assess the sensitivity and representativeness of leishmaniasis surveillance in Spain, being a useful tool to assess whether the generalization of leishmaniasis surveillance throughout the Spanish territory achieves a reduction in underreporting.

## Introduction

Leishmaniasis is a parasitic disease caused by protozoa of the genus *Leishmania* and transmitted by the bite of infected sandflies of the genera *Phlebotomus* (Old World) and *Lutzomyia* (New World). Natural transmission may be zoonotic or anthroponotic. Worldwide, at least 20 *Leishmania* species are causing leishmaniasis. Three clinical forms are known: cutaneous leishmaniasis (CL), causing skin sores and eventually scars, systemic or visceral leishmaniasis (VL) that can lead to deadly complications if left untreated, and mucocutaneous leishmaniasis (MCL), causing destruction of mucous membranes [1, 2].

In Europe, human leishmaniasis is endemic in the Mediterranean basin, with two endemic cycles of transmission: the zoonotic form of CL and VL caused by *L*. *infantum* throughout the Mediterranean region and the anthroponotic form of CL caused by *L*. *tropica*, with occasional occurrence in Greece and neighbouring countries [3]. In 2017, the incidence of VL and CL in Europe was estimated at less than 2% of the disease‘s global burden, with almost 75% of the cases concentrated in Albania, Georgia, Italy and Spain [4]. Currently, in Europe there is an expansion towards northern latitudes, especially in endemic countries such as Spain or Italy, so it can be considered as an emerging disease [5].

In Spain, leishmaniasis is endemic in most of the Iberian Peninsula and the Balearic Islands. Autochthonous cases are caused by *L*. *infantum*. The dog is the main domestic reservoir, although wild mammals have been also found infected with *L*. *infantum*. The vectors involved in transmission are *Phlebotomus pernicious and P*. *ariasi* [3, 6]. Leishmaniasis is a communicable disease (CoD) since 1982. Between 1996 and 2014, due to the decentralization of the surveillance system, it was monitored only by those autonomous regions (CCAA in Spanish) where it was considered endemic. In 2009, the largest outbreak of leishmaniasis known to date in the Mediterranean basin started in Madrid [7]. This outbreak revealed the existence of underreporting, already suspected from previous studies [8–10]. It also highlighted the importance of monitoring the disease throughout the country. Consequently, since 2015, leishmaniasis was considered again a CoD throughout the territory [11].

From 2005 to 2017, 2,733 new cases of leishmaniasis were reported to the National Surveillance Network (RENAVE) and, considering only those CCAA with stable notification, the range of the annual incidence rate (IR) was 0.30 cases per 100,000 inhabitants (years 2008 and 2009) to 1.06 (2011), being the media IR for the studied period of 0.62 [11].

This study aimed at estimating the incidence of leishmaniasis and to evaluate the extent of underreporting for 2016 and 2017 by the RENAVE in Spain, using the capture-recapture (CRC) method.

## Methods

### Data sources and case definitions

Two sources of data were used to estimate the IR of leishmaniasis in Spain. The first source was RENAVE registers for confirmed or probable cases of leishmaniasis. The second data source, the Hospital Discharge Records of the National Health System (CMBD), includes hospitalizations related to leishmaniasis.

RENAVE was established in 1995 with the aim of collecting and analysing epidemiological information that allows detecting problems that pose a risk to the public health. It integrates the notification and epidemiological investigation of cases of communicable diseases, outbreaks or microorganisms. The notification is made following guidelines or protocols agreed by all the members of RENAVE (representatives of the CCAA, Carlos III Health Institute and the Ministry of Health, Consumption and Social Welfare ‒MSCBS‒), which are periodically reviewed and updated [12]. Surveillance is comprehensive since 2015, as it should include cases detected from all territories and all sectors (public and private health facilities or laboratories). Probable and confirmed cases of leishmaniasis are reported weekly by the regional epidemiological surveillance networks to the National Centre for Epidemiology through RENAVE, and the information is annually consolidated [3]. Collected information includes autonomous region and province (residence and place of infection), date of birth, sex, age, case classification, date of diagnosis, date for statistics (date of symptoms onset when available or the closest one), clinical category, origin (imported or not), hospitalization and death. The variable ‘date of the case’ was created for RENAVE (date closest to the date of hospitalization / discharge, according to CMBD), assigning it the diagnosis or the date for statistics if that was not available.

In the RENAVE dataset, a case is considered as probable when meets the clinical criteria for CL, MCL or VL and there is an epidemiological link (HIV infection or contact with an infected animal). A case is considered confirmed when it meets the criteria of probable case and is laboratory confirmed (detection of the parasite by microscopy or culture, or its DNA by PCR; or a positive serology for VL) [3].

CMBD is a set of standardized clinical-administrative data of hospitalizations that allows knowing the morbidity attended in public and private hospitals. It was approved by the Interterritorial Council of the National Health System in 1987. In 2016, the Register of Specialized Health Care Activity (RAE-CMBD) was implemented as a new data model [13]. In RAE-CMBD, up to 20 diagnostic variables are recorded, corresponding to those diagnostics done during hospitalization, which are coded following the International Classification of Diseases (ICD-10-ES). In this study, hospitalizations coded as leishmaniasis (codes B55.0- Visceral leishmaniasis; B55.1 –Cutaneous leishmaniasis; B55.2 –Mucocutaneous leishmaniasis; B55.9 –Leishmaniasis, unspecified) in any diagnostic position were selected. Information included the date of birth, age, sex, date of hospitalization, main and secondaries diagnosis, clinical classification, outcome, autonomous region, province and postal code of residence.

The population studied comprises all the cases that were registered in at least one of these information systems in 2016 and 2017, after data preparation: removal of duplicities and rehospitalizations (CMBD generates a record of each hospital discharge, so patients requiring multiple hospitalizations are recorded each time; however, RENAVE generates a single record per patient).

### Data analysis

Data for 2016 and 2017 were cleaned, selecting the probable and confirmed leishmaniasis cases from RENAVE and the first hospital admission (incident hospitalization) of the leishmaniasis cases from CMBD.

A descriptive analysis was performed according to the clinical classification of leishmaniasis, and the frequencies and percentages by sex, age groups and outcome were calculated. Within each data source, a comparison was made between the clinical forms according to sex, age and death, using Pearson’s χ2 test or Fisher’s exact test if necessary, considering statistically significant differences those with p <0.05.

Through the CRC analysis, an estimation of the incidence of human leishmaniasis in Spain and the sensitivity of the information systems analysed was performed. The CRC method is an indirect method to estimate incidence from two or more independent data sources. It uses statistical models of the degree of overlap between independent data sets to calculate those that are not represented in the available records [14]. This method arose in the field of ecology to obtain censuses of wild and fish populations when only a small proportion of the individuals of the population to be studied can be detected [15].

The data provided by RENAVE were compared with the CMBD records. The CRC study was carried out according to the clinical form. As there is not a unique common identifier between both data registers, a combination of variables was used to match cases in both data sources (Table 1).

**Table 1:** Overlapping variables.

RENAVE	CMBD
Year	Year
Autonomous Region of residence	Autonomous Region of residence
Province of residence	Postal Code
Date of birth	Date of birth
Sex	Sex
Age	Age
Month of the case	Month of discharge
Clinical category	Clinical classification

CMBD: Hospital Discharge Records of the National Health System; RENAVE: National Surveillance Network.

The modified Chapman [16] and Seber [17] formula was used to calculate the number (N) of cases of leishmaniasis in the population, with the 95% confidence interval (95% CI) for the years 2016 and 2017, using the formulas bellow:

$$N=\frac{\left(M+1)(n+1\right)}{m+1}-1$$

$$IC\;95\%=N\pm 1.96\sqrt{Var}(N)$$

$$Var(N)=\frac{\left(M+1)(n+1)(M-m)(n-m\right)}{\left(m+1)(m+1)(m+2\right)}-1$$

Where N is the estimate of the number of cases; M the number of cases from the first source; n the number of cases from the second source and m the number of common cases.

The completeness rate for each of the 2 records reviewed was calculated according to the formula:

$$S1=\frac{M}{N}\times 100$$

$$S2=\frac{n}{N}\times 100$$

And the completeness rate for the 2 records combined:

$$S1y2=\frac{M+n-m}{N}\times 100$$

The hospitalizations and notified cases with a defined clinical form were included (we eliminated cases with B55.9 code in CMBD and the missing observations at clinical category in RENAVE). The RENAVE records with unknown date of birth were modified (matching the records by: community of residence, age, sex and month of discharge, and then replacing this data from the information obtained from the CMBD, only if: i) they matched on these variables at 100%; ii) the province of residence did not differ; and iii) the CMBD hospitalization was not paired with another reported case that had date of birth).

For the CRC study, we used the software EPIDAT, version 3.1 [18]. The program assumes that the two sources are independent and estimates the total number of cases, with their confidence interval (CI), as well as the completeness of each of the lists separately and jointly [19].

For the calculation of IR, the population at risk was estimated at 44,679,476 and 44,768,038 (data from the census population projections as of January 1, 2016 and 2017, respectively, calculated by the National Institute of Statistics were used [20]).

### Ethical statement

This study involves the use of patient medical data from RENAVE and CMBD. Both are official sources which follow the mandate of Spanish and international legislation and are hosted by public institutions. Therefore, data are available under request to the Ministry of Health and National Centre for Epidemiology, including the justification of the request and a confidentiality agreement, with the commitment not to use share data with third parties. Individual informed consent is not required for data to be included in RENAVE and CMBD, and all data are pseudonymized, meeting all considerations regarding personal data protection. As this work is in line with surveillance activities, no explicit ethics assessment was required.

## Results

### Sociodemographic and clinical characteristics of reported cases and hospitalizations for leishmaniasis

**Fig 1:**
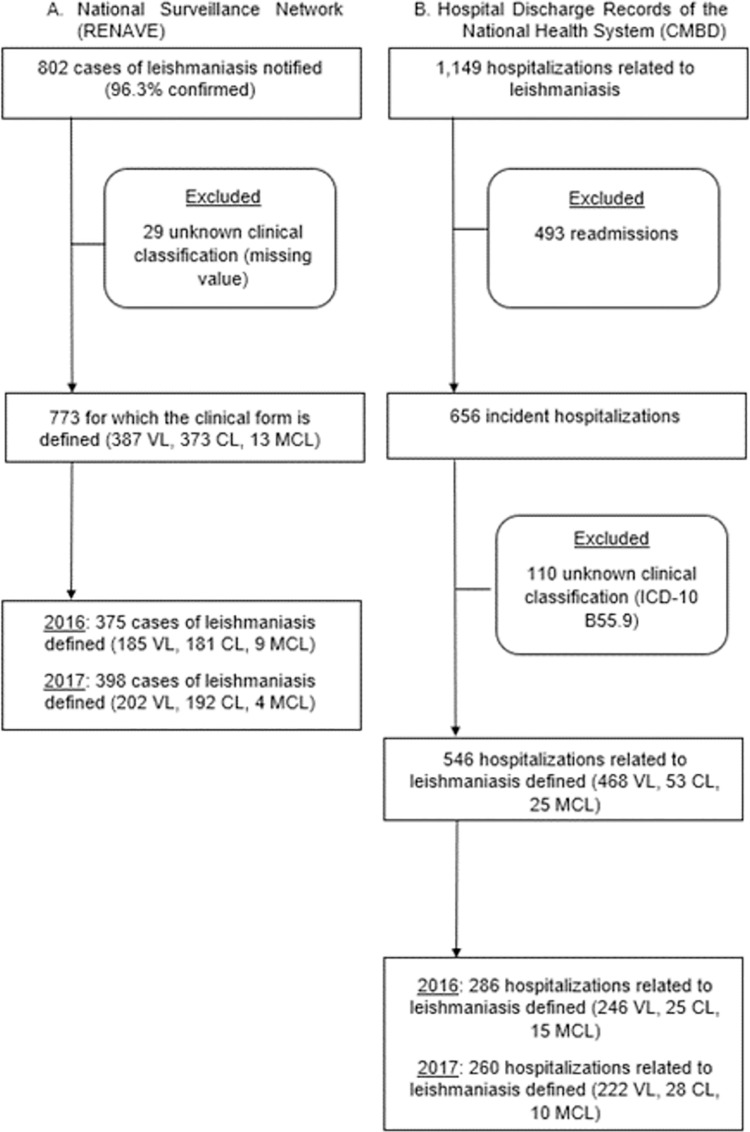
Extraction of cases of leishmaniasis from the registries at RENAVE and CMBD, Spain, 2016–2017. ICD: International Classification of Diseases; CL: Cutaneous Leishmaniasis; MCL: Mucocutaneous Leishmaniasis; VL: Visceral Leishmaniasis.

Between 2016 and 2017, 802 cases of leishmaniasis were notified to RENAVE. 96.3% of them (n = 772) were confirmed cases. The reported cases with defined clinical classification of leishmaniasis were 773 (96.4%). In the same period, 1,149 hospitalizations related to leishmaniasis were registered in CMBD along the Spanish territory. After eliminating all readmissions, the number of incident hospitalizations was 656, of which 546 (83.2%) had a defined clinical classification (Fig 1).

Considering only the cases with known clinical classification (n = 773; 96.4% confirmed) reported to RENAVE from 2016–2017, it was observed that in VL and CL people between 45–65 years old predominated, while in MCL, those over 65. Out of all female cases, CL was the most common manifestation (53%) (p <0.01). The percentage of hospitalization was higher in VL (235/425; 94.4%). Most deaths occurred in this group (only 1 death was registered for MCL) (Table 2).

**Table 2:** Sociodemographic characteristics of leishmaniasis cases according to the data source, RENAVE and CMBD, Spain, 2016–2017.

		RENAVE						CMBD						
		Clinical classification (n = 773)						Clinical classification (n = 546)						
		VL		CL		MCL		VL		CL		MCL		
		n	%	n	%	n	%	n	%	n	%		n	%
		387	50.1	373	48.2	13	1.7	468	85.7	53	9.7		25	4.6
Sex	Man	255	66.1	221	59.2	9	69.2	320	68.4	37	69.8		19	76
	Woman	131	33.9	152	40.7	4	30.8	148	31.6	16	30.2		6	24
Age (years)	<1	32	8.3	28	7.5	0	-	34	7.3	4	7.5		0	-
	1–4	65	16.8	31	8.3	0	-	64	13.7	2	3.8		0	-
	5–14	10	2.6	41	11	0	-	17	3.6	3	5.7		1	4
	15–44	79	20.4	65	17.4	0	-	101	21.6	13	24.5		2	8
	45–64	107	27.6	130	34.8	5	38.5	161	34.4	13	24.5		12	48
	≥65	94	24.3	78	20.9	8	61.5	91	19.4	18	34		10	40
Death	No	204	93.1	153	100	3	75	442	94.4	51	96.2		25	100
	Yes	15	6.8	0	-	1	25	26	5.6	2	3.8		0	-

CL: Cutaneous Leishmaniasis; CMBD: Hospital Discharge Records of the National Health System; MCL: Mucocutaneous Leishmaniasis; VL: Visceral Leishmaniasis; RENAVE: National Surveillance Network.

Among CMBD hospitalizations with a defined clinical classification of leishmaniasis (n = 546), VL predominated in both men (85.1%) and women (87.1%). Like in RENAVE, 45-65-years-old-group predominated in VL. However, in CL over 65 years of age were more frequent (p <0.01). The percentage of deaths was higher in VL (Table 2).

In these years, according to RENAVE, the IR was 0.4–0.43, 0.39–0.41 and 0.01–0.02 per 100,000 inhabitants for VL, CL and MCL, respectively. The hospitalization IR of VL was 0.48–0.53, CL 0.05–0.06 and MCL 0.02–0.03.

### Capture–recapture analysis

**Fig 2:**
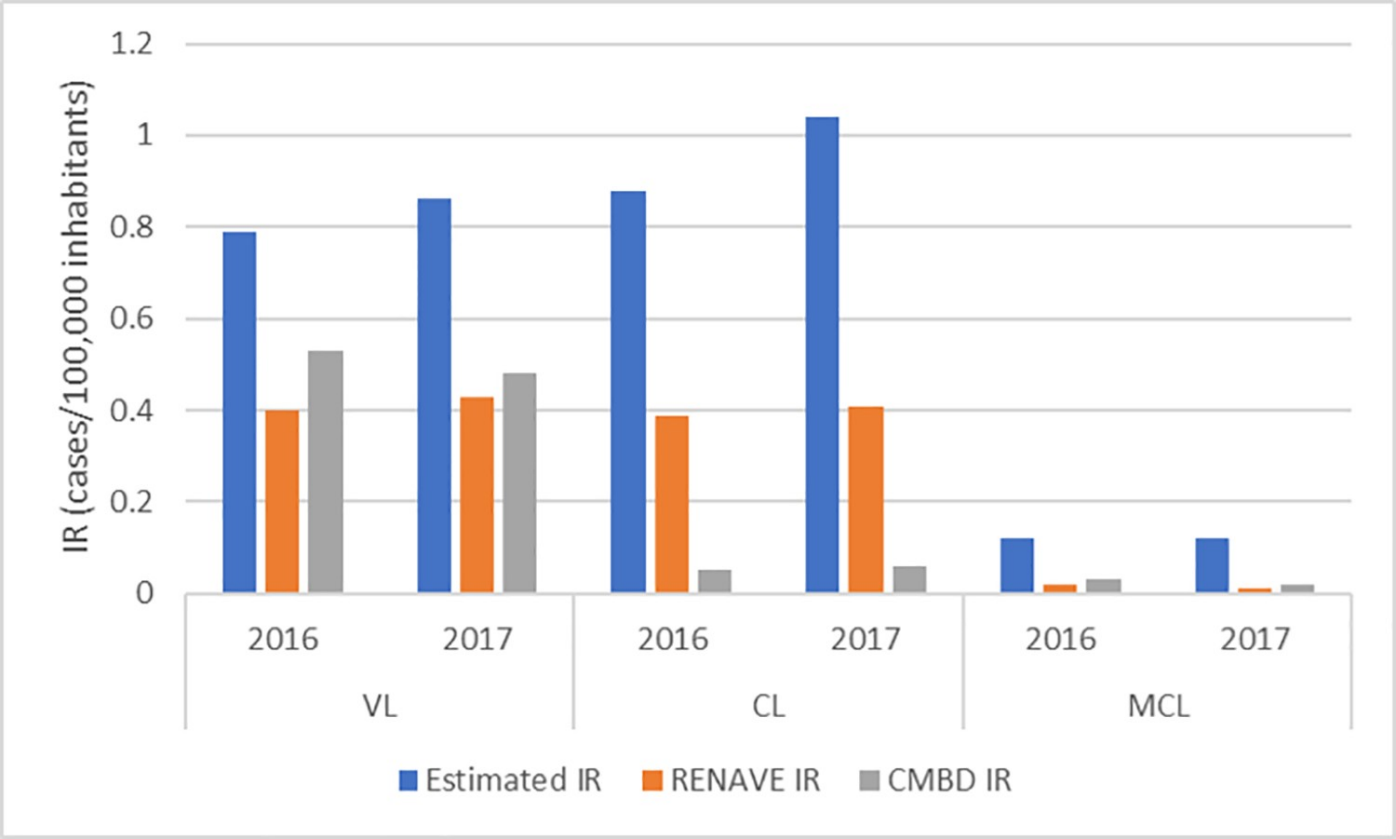
Estimated IR of human leishmaniasis according to the CRC method, Spain, 2016–2017. CL: Cutaneous Leishmaniasis; CMBD: Hospital Discharge Records of the National Health System; CRC: Capture-Recapture; IR: Incidence Rate; MCL: Mucocutaneous Leishmaniasis; VL: Visceral Leishmaniasis; RENAVE: National Surveillance Network.

In 2016, 431 cases of VL were identified by the two data sources (185 by RENAVE and 246 by CMBD) (Table 3). 128 of the total cases were captured by the two data sources. The total number of affected people was estimated to be 355 (95% CI: 332–378) with an IR of 0.79 per 100,000 inhabitants (Fig 2). Regarding the exhaustiveness of these registries, CMBD detects 69.3% of the VL cases that are estimated, while RENAVE detects 52.1%; considering both registries, underreporting of VL was estimated to be 14.7%.

**Table 3:** Number of cases of leishmaniasis, by data source, after case-linkage, and combined estimates based on CRC analysis, Spain, 2016–2017.

Year	Clinical classification	Registered number of unique cases per database		Combined data			CRC analysis	% under-reporting RENAVE	% under-reporting CMBD	% under-reporting RENAVE + CMBD
		RENAVE M	CMBD n	RENAVE only	CMBD only	RENAVE-CMBD m	Total N_estimated_ (95% CI)			
2016	VL	185	246	57	118	128	355 (332–378)	47.9%	30.7%	14.7%
	CL	181	25	170	14	11	393 (242–545)	54%	93.4%	50.4%
	MCL	9	15	7	13	2	52 (13–91)	82.8%	71.3%	58%
2017	VL	202	222	86	106	116	385 (355–417)	47.7%	42.5%	20.2%
	CL	192	28	181	17	11	465 (278–653)	58.7%	94%	55.1%
	MCL	4	10	4	10	0	54 (-10-119)	92.6%	81.5%	74.1%

CI: confidence interval; CMBD: Hospital Discharge Records of the National Health System; M: number of cases registered by RENAVE; n: number of cases registered by CMBD; m: number of cases registered in common in RENAVE and CMBD databases; N_estimated_: estimated number of cases; RENAVE: National Surveillance Network.

The capture–recapture estimates are calculated separately for each calendar year (2016 and 2017).

In the same year, 206 cases of CL and 24 of MCL were registered together by RENAVE and CMBD. The total number of affected people was estimated at 393 for CL (95% CI: 242–545) and 52 (95% CI: 13–91) for MCL. The IRs were 0.88 (CL) and 0.12 (MCL), representing an underreporting of 50.4% and 58% for CL and MCL, respectively. RENAVE detects 46% of CL cases and 17.2% of MCL cases that are estimated, while CMBD detects 6.4% and 28.7%, respectively.

In 2017, 424 cases of VL were identified, 116 in both CMBD and RENAVE (Table 3). The total number of affected people was estimated at 385 (95% CI: 355–417) with an IR of 0.86 and an underreporting of 20.2%. In the same year, 220 cases of CL and 14 of MCL were registered. The total number of affected people was estimated at 465 for CL (95% CI: 278–653) and 54 (95% CI: -10-119) for MCL. The IRs were 1.04 (CL) and 0.12 (MCL). According to these estimates, 55.1% of CL cases and 74.1% of MCL were not registered. RENAVE detects 52.3% of VL, 41.2% of CL cases and 7.4% of MCL cases that are estimated, while CMBD detects 57.5%, 6% and 18.5%, respectively.

Considering all cases for both years and both registries, underreporting of VL was estimated to be 17.6%, CL 53.9% and MCL 70%.

## Discussion

Our results support the existence of underreporting of cases of leishmaniasis and quantify its magnitude, by estimating an IR of the disease higher than that reflected by RENAVE in 2016–2017 in Spain [11]. In addition, the application of the capture-recapture method to compare the IR obtained by RENAVE and CMBD has allowed us to assess the completeness of both records, being greater in RENAVE, as expected if we bear in mind that CMBD only record leishmaniasis cases requiring hospitalization and also that the difference in the completeness of VL between the two sources is not large compared to that of CL.

Both data sources are geographically comprehensive as they include cases form every autonomous Spanish region. We consider them to be complementary, as RENAVE includes notifications from all health care facilities (primary care and hospitalizations) and CMBD collects all cases discharged with leishmaniasis diagnostic, many of them maybe not actively notified to RENAVE.

Although this method has been applied in other countries to analyse leishmaniasis incidence, such as Brazil, Argentina and Bolivia [21–23], it is the first time, to our knowledge, that it has been used in a country belonging to the WHO European region. Nevertheless, this type of study has been useful at the European level to estimate the incidence of other infections that cause a large burden of disease such as hepatitis C or malaria [24, 25].

Our results for both RENAVE+CMBD show an improvement in reporting compared to previous publications, which estimated an underreporting of 25–40% for VL and almost 100% for CL in Spain [3], although the underreporting in RENAVE, which is actually the national surveillance data source, is higher. In countries like Bolivia and Brazil, this method found underreporting percentages of around 73% for CL in Bolivia and 42–45% for VL in Brazil [21, 23]. The use of standardized and validated methods to evaluate a surveillance system would allow for more reliable comparisons over time and between different countries and should be included among the routine tasks of surveillance activities.

We have estimated a much higher incidence of leishmaniasis in Spain than in neighbouring countries such as France (1999–2012) and Greece (1981–2011), where an average annual IR is estimated at 0.22 and 0.36 per 100,000 inhabitants during the mentioned periods, respectively [26, 27]. However, comparisons between countries and in different timeframes are of limited value if not accompanied by an estimation of the magnitude of underreporting in those other countries by using similar methods. Moreover, despite being endemic in the Mediterranean basin, there are not standardised criteria for leishmaniasis surveillance in Europe (nor case definition for surveillance purposes using either surveillance method). In addition, it is a notifiable disease in some countries but from different approaches: from comprehensive surveillance (Greece, Italy, Turkey, Spain), or sentinel from selected laboratories in others (France), or even not under surveillance in others. As a result, surveillance data are fragmented in Europe and comparisons are challenging [2].

The higher percentage of underreporting of CL/MCL versus VL may be due to the fact that VL requires hospitalization more frequently [9], with more tests being performed in hospitals leading to its final diagnosis; in contrast, CL usually resolves without treatment. On the other hand, public health hospital services are usually more familiar with the reporting procedures of the CoD than the primary health care centres [28], even if the notification is automatic from the computerized medical record in some CCAA (suspected CoD cases according to ICD-10 codes are imported directly from the digital medical record to the surveillance network) [29]. In addition, patients with milder forms (CL) may seek health care to a lesser extent and the etiological diagnosis is not always reached due to its complexity, the difficulty in differential diagnosis with other skin pathologies, or to the lack of diagnostic tests [30]. However, we must bear in mind that due to the low number of reported MCL cases both at RAE-CMBD and RENAVE, these estimations should be interpreted with caution.

Other factors such as lack of time and overwork, lack of awareness of the importance of notification, maintenance of patient confidentiality, lack of feedback from public health services and preventive medicine services and lack of knowledge regarding the mandatory reporting diseases are among other reasons for underreporting by health professionals [28].

The differences observed in the percentages of underreporting between 2016 and 2017 could be due to the unequal completion of the data recorded during these years. In a study analysing data for 2014–2017, a deficit in the RENAVE completeness, with a wide margin for improvement in the quality of the information provided by the CCAA, as well as in the variables included in the registry and the way they were collected, was found [11].

Among the limitations of this study, the variability in the data coding according to the quality of the medical discharge report (CMBD) or the notification questionnaire (RENAVE) stands out. The greatest difficulty in comparing both datasets is the absence of a unique identifier, although an algorithm combining a sequence of coincident variables was created to match cases. A key assumption of the two-source capture-recapture method is the independence of sources. However, in human populations, it is highly unlikely to have complete independence between sources [19, 31]. In our case, both sources are independent a priori, although probably those cases that require hospitalization are more prompted to be reported into RENAVE. In fact, some autonomous communities use CMBD to capture cases of CoD that have not been reported, in addition to the computerized clinical history in primary care and laboratory records. Although the correction remains imperfect if notifications from both sources are positively dependent, underestimation of case numbers is typically much less severe than with the traditional registration approach. On the other hand, the degree of overlap between the databases is largely due to the quality of completion of the data, so that the existence of missing values (clinical form in CMBD, to which are also added in RENAVE hospitalization and death) may have underestimated the number of coincident cases in both sources.

Despite this, CMBD and RENAVE have numerous advantages, among which the accessibility and availability of the data stand out, which allow us to respond to the growing demand for health information at no cost. While CMBD is a representative clinical-administrative database of the population requiring admission, the RENAVE is created for surveillance purposes and collects incident cases of leishmaniasis, which are representative of the general population. Therefore, we believe that the estimate made through the capture-recapture method is the best approximation that can be made with the existing information.

The stablishing of leishmaniasis as a notifiable disease in the whole country may have had a positive impact on a better understanding of the epidemiology of the disease. Nevertheless, it is still necessary to improve its completeness.

The evaluation of a surveillance system promotes the best use of public health resources and help improve our understanding of infectious diseases epidemiology [32] and should be included among the routine activities of a public health surveillance system. In this case, the CRC method has helped us to assess the sensitivity and representativeness of leishmaniasis surveillance in Spain. The CRC methodology might be a useful tool to assess whether the generalization of leishmaniasis surveillance throughout the Spanish territory achieves a reduction in underreporting. In fact, we are planning to apply this system with more recent years as the data become available to evaluate this reduction, as well as in the different CCAA since the sensitivity in case detection may vary according to the region.

Finally, to strengthen the epidemiological surveillance of leishmaniasis, it would be necessary to increase the awareness of health authorities and political decision-makers, and also the health and public health professionals for the diagnosis and reporting of the disease, allowing a rapid response by surveillance systems to stop its expansion. In the same way, making the population aware of the existence of preventive measures to avoid the spread of the disease would help to reduce its burden. Last but not least, providing that leishmaniasis is a zoonosis, the better the notification is, the more targeted and appropriate the control strategies can be. The control of vectors and animal reservoirs are the best measure to reduce transmission. Therefore, it is desirable to have strategies or plans with a comprehensive approach ("One Health") that bring together all aspects of the disease: both surveillance in humans and reservoirs, entomological and environmental, and review the preventive measures to be applied according to the different risk scenarios, achieving an improvement in the sensitivity of the surveillance system.
